# Full-Arch Implant Rehabilitation Using Multi-Unit Abutments in a Completely Edentulous Patient: A Clinical Case Report

**DOI:** 10.7759/cureus.107595

**Published:** 2026-04-23

**Authors:** Arvind K Singh, Mohit Raghuvanshi, Shalini Pandey, Ahustosh P Singh, Kshitij Chaturvedi

**Affiliations:** 1 Department of Prosthodontics, Chandra Dental College and Hospital, Barabanki, IND; 2 Department of Conservative Dentistry and Endodontics, Chandra Dental College and Hospital, Barabanki, IND; 3 Department of Oral and Maxillofacial Surgery, Maharana Pratap College of Dentistry and Research Centre, Gwalior, IND

**Keywords:** dental implants, full-arch rehabilitation, implant-supported prosthesis, multi-unit abutments, osseointegration, prosthodontics

## Abstract

Implant-supported prostheses are a predictable treatment option for the rehabilitation of completely edentulous patients. When appropriate surgical and prosthodontic protocols are followed, they effectively restore function, esthetics, and occlusal stability. This case report describes the management of a 50-year-old male patient with complete maxillary and mandibular edentulism using implant-supported full-arch prostheses with multi-unit abutments (MUAs).

Clinical and radiographic evaluation, including orthopantomogram and cone beam computed tomography, was performed for treatment planning. A total of 12 endosseous implants were placed, six in each arch, achieving a primary stability of 35 Ncm. Prosthetic rehabilitation was carried out using an open-tray impression technique, accurate jaw relation records, and passive framework verification. A screw-retained definitive prosthesis was subsequently delivered.

All implants demonstrated successful osseointegration with satisfactory stability. The final prostheses exhibited good esthetics, functional occlusion, and improved patient comfort. The patient reported enhanced mastication and overall satisfaction.

This case highlights that full-arch implant rehabilitation using screw-retained prostheses with MUAs is a reliable and predictable treatment approach for completely edentulous patients.

## Introduction

Complete edentulism remains a significant clinical challenge, often leading to compromised masticatory function, impaired phonetics, and reduced quality of life. Implant-supported full-arch prostheses have emerged as a predictable and well-documented treatment modality for the rehabilitation of edentulous patients, offering superior stability, retention, and patient satisfaction compared to conventional removable prostheses [1,2]. The number and distribution of implants play a critical role in determining the biomechanical success of full-arch restorations, with fixed prostheses typically supported by five to ten implants depending on anatomical and functional considerations [3]. Among these configurations, the “all-on-six” concept has gained increasing acceptance due to its favorable load distribution, enhanced prosthetic support, and reduced risk of mechanical complications.

In clinical scenarios with adequate bone volume and favorable arch form, the selection of an all-on-six configuration with multi-unit abutments (MUAs) provides improved biomechanical stability, reduced cantilever length, and enhanced prosthetic support compared to reduced implant protocols such as all-on-four. However, there remains a need to clearly delineate the clinical decision-making behind selecting an all-on-six approach in specific anatomical contexts, particularly where sufficient bone availability permits optimal implant distribution and prosthetically driven planning. The present case addresses this gap by demonstrating the rationale and clinical outcomes of using six implants per arch with MUAs in a fully edentulous patient.

The introduction of MUAs has significantly advanced the restorative phase of implant dentistry by facilitating screw-retained prosthetic designs and compensating for discrepancies in implant angulation. These abutments serve as intermediaries between implants and prostheses, facilitating passive fit and improving stress distribution across the prosthetic framework [4,5]. Achieving a passive and accurate fit is essential, as a misfit at the implant-prosthesis interface has been associated with biological complications, such as marginal bone loss, and mechanical failures, including screw loosening and component fracture [6].

Radiographic evaluation using cone-beam computed tomography (CBCT) plays a crucial role in implant planning by allowing precise assessment of bone volume, density, and anatomical limitations, including sinus morphology and available bone height [7].

Full-arch implant rehabilitation with MUAs enables the fabrication of retrievable, screw-retained prostheses that enhance maintenance and long-term clinical outcomes. Furthermore, the use of six implants per arch provides improved biomechanical stability and minimizes cantilever forces, particularly in cases with adequate bone volume, thereby contributing to the longevity of the restoration [2,3]. Soft-tissue management, including appropriate flap design and suturing techniques, is essential to ensure optimal healing and peri-implant tissue stability [8]. Additionally, postoperative protocols, including antimicrobial prophylaxis and oral rinses, play an important role in minimizing complications and enhancing patient recovery [9].

This clinical case report presents the prosthetic rehabilitation of a completely edentulous patient using implant-supported full-arch prostheses with six implants per arch and MUAs, highlighting the clinical workflow, prosthetic considerations, and treatment outcomes.

## Case presentation

A 50-year-old male patient presented to the Department of Prosthodontics with complaints of compromised masticatory efficiency and dissatisfaction with existing removable complete dentures. The patient reported difficulty in chewing fibrous foods, reduced prosthesis stability, and decreased comfort, adversely affecting his quality of life. Medical history was non-contributory, with no contraindications for implant therapy. Extraoral examination was unremarkable. An intraoral examination revealed completely edentulous maxillary and mandibular arches with adequate ridge height, width, and favorable morphology. The mucosa was healthy, and sufficient inter-arch space was available. The existing dentures demonstrated poor retention, stability, and occlusal harmony, warranting definitive rehabilitation.

**Figure 1:**
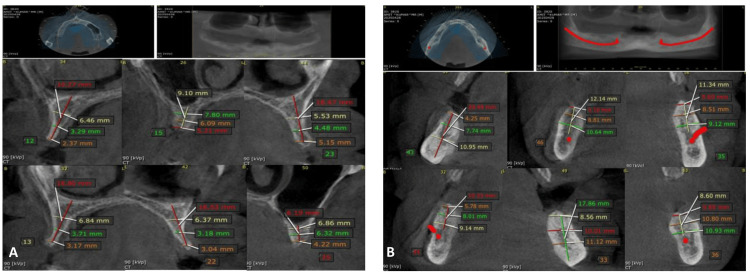
Preoperative radiographic assessment A: Cone-beam computed tomography (CBCT) images of the maxillary arch demonstrating cross-sectional views at planned implant sites, with measurements of ridge width, bone height, and angulation relative to the maxillary sinus. Arrows and reference lines indicate implant trajectory and proximity to anatomical structures; B: CBCT images of the mandibular arch showing cross-sectional views at implant sites, illustrating bone dimensions and angulation in relation to the inferior alveolar nerve and mental foramen. Color-coded measurements represent ridge width, available bone height, and safety margins.

Radiographic evaluation using CBCT was performed for prosthetically driven planning. The anterior maxilla and mandible showed favorable bone availability, while the posterior maxilla required angulation considerations due to proximity to the maxillary sinus [7]. In the mandible, the inferior alveolar nerve and mental foramina were clearly identified, ensuring safe implant placement. Based on combined clinical and radiographic assessment, the edentulous ridges were classified as Cawood and Howell Class IV [10]. Overall, the findings supported full-arch implant rehabilitation without the need for extensive bone augmentation (Figures 1A, 1B).

Following comprehensive clinical and radiographic evaluation, a prosthetically driven implant placement protocol with a delayed loading protocol was planned for full-arch rehabilitation of both maxillary and mandibular arches. Written informed consent was obtained from the patient prior to the surgical procedure.

All surgical procedures were performed under strict aseptic conditions. Preoperative antimicrobial prophylaxis was administered, and the patient was instructed to rinse with 0.2% chlorhexidine gluconate solution for one minute prior to surgery. Local anesthesia was achieved using 2% lignocaine with 1:80,000 epinephrine.

**Figure 2:**
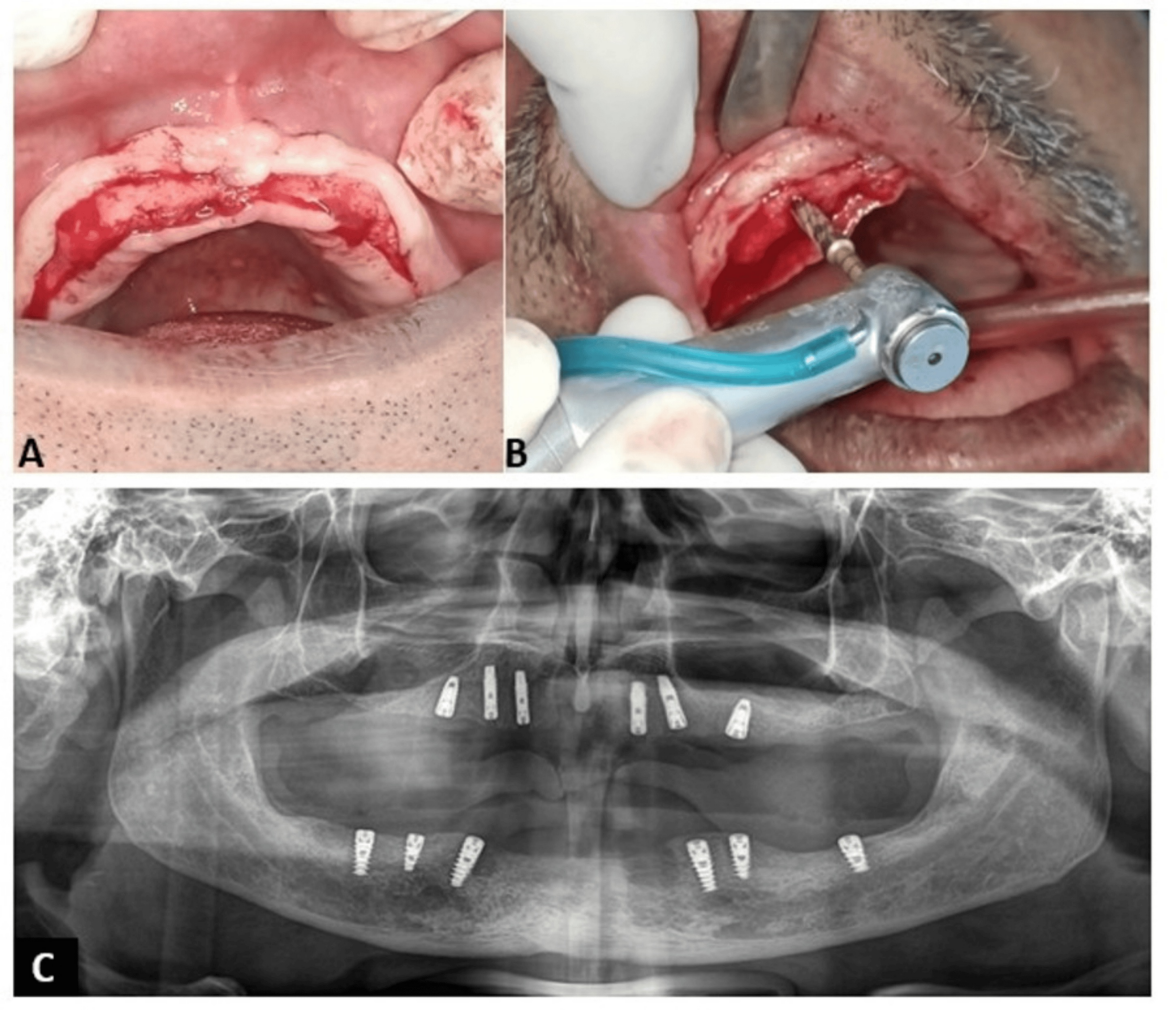
Surgical phase of implant placement and postoperative radiographic evaluation A: Mid-crestal incision in the maxillary arch with reflection of a full-thickness mucoperiosteal flap, exposing the underlying alveolar ridge for implant placement; B: Osteotomy preparation using a Densah bur in densifying mode, demonstrating the osseodensification technique to enhance bone density and primary stability in the maxilla; C: Postoperative orthopantomogram (OPG) showing the position, angulation, and distribution of implants in both arches, confirming adequate anteroposterior spread and avoidance of critical anatomical structures

A mid-crestal incision with minimal vertical releasing incisions was made in both arches to reflect a full-thickness mucoperiosteal flap, allowing adequate visualization of the underlying alveolar ridge (Figure 2A). In the maxillary arch, osteotomy preparation was performed using osseodensification burs (Densah burs) in a counterclockwise (densifying) mode. This technique facilitated preservation and compaction of bone along the osteotomy walls, thereby enhancing bone density and improving primary implant stability, particularly in the relatively softer maxillary bone (Figure 2B). Care was taken to maintain appropriate angulation to avoid the maxillary sinus and to optimize anterior-posterior spread. In the mandibular arch, conventional sequential drilling was performed, ensuring precise implant bed preparation while maintaining safe distances from critical anatomical structures. 

A total of 12 endosseous implants were placed, six in each arch, strategically distributed to achieve optimal biomechanical support and load distribution. The implants were positioned in a prosthetically driven manner, with anterior implants placed axially and posterior implants angled where necessary to maximize anteroposterior spread and reduce cantilever length. Primary stability was achieved in all implants, with insertion torque values of approximately 35 Ncm.

In the maxillary arch, implants from the DIO Implant System (DIO Implant Co., Busan, South Korea) were placed at positions 12, 22, and 13 (3.3 × 11.5 mm), 23 (3.8 × 11.5 mm), 15 (4.0 × 8.5 mm), and 26 (4.0 × 8.5 mm). In the mandibular arch, implants from the ADIN Implant System (ADIN Dental Implant Systems Ltd., Afula, Israel) were placed at positions 43 (4.2 × 11.5 mm), 45 (3.75 × 8 mm), 46 (4.2 × 10 mm), 33 (3.75 × 11.5 mm), 35 (3.75 × 10 mm), and 36 (4.2 × 8 mm). Following implant placement, cover screws were placed as per the planned loading protocol. The surgical sites were irrigated with sterile saline, and a full-thickness mucoperiosteal flap was repositioned and sutured using non-resorbable sutures to achieve primary closure [8]. A postoperative panoramic radiograph was obtained to verify the position, angulation, and distribution of implants in both arches (Figure 2C).

Following a healing period of five months, in accordance with a delayed loading protocol, second-stage surgery was performed to expose the implants in both arches. Under local anesthesia, a crestal incision was made, and the implants were uncovered. Subsequently, MUAs of appropriate collar heights and angulations were selected and connected to the implants to achieve optimal prosthetic platform positioning and maintain parallelism across the arch. The use of MUAs aided in the correction of implant angulation discrepancies and established a common path of insertion, which is critical for full-arch screw-retained prostheses. In the maxillary arch (DIO Implant System), straight (0°) abutments were used at positions 12, 13, and 22, while angulated abutments of 17° were placed at positions 23 and 30° abutments at positions 15 and 26 to achieve prosthetic alignment. In the mandibular arch (ADIN Implant System), all MUAs were straight (0°).

**Figure 3:**
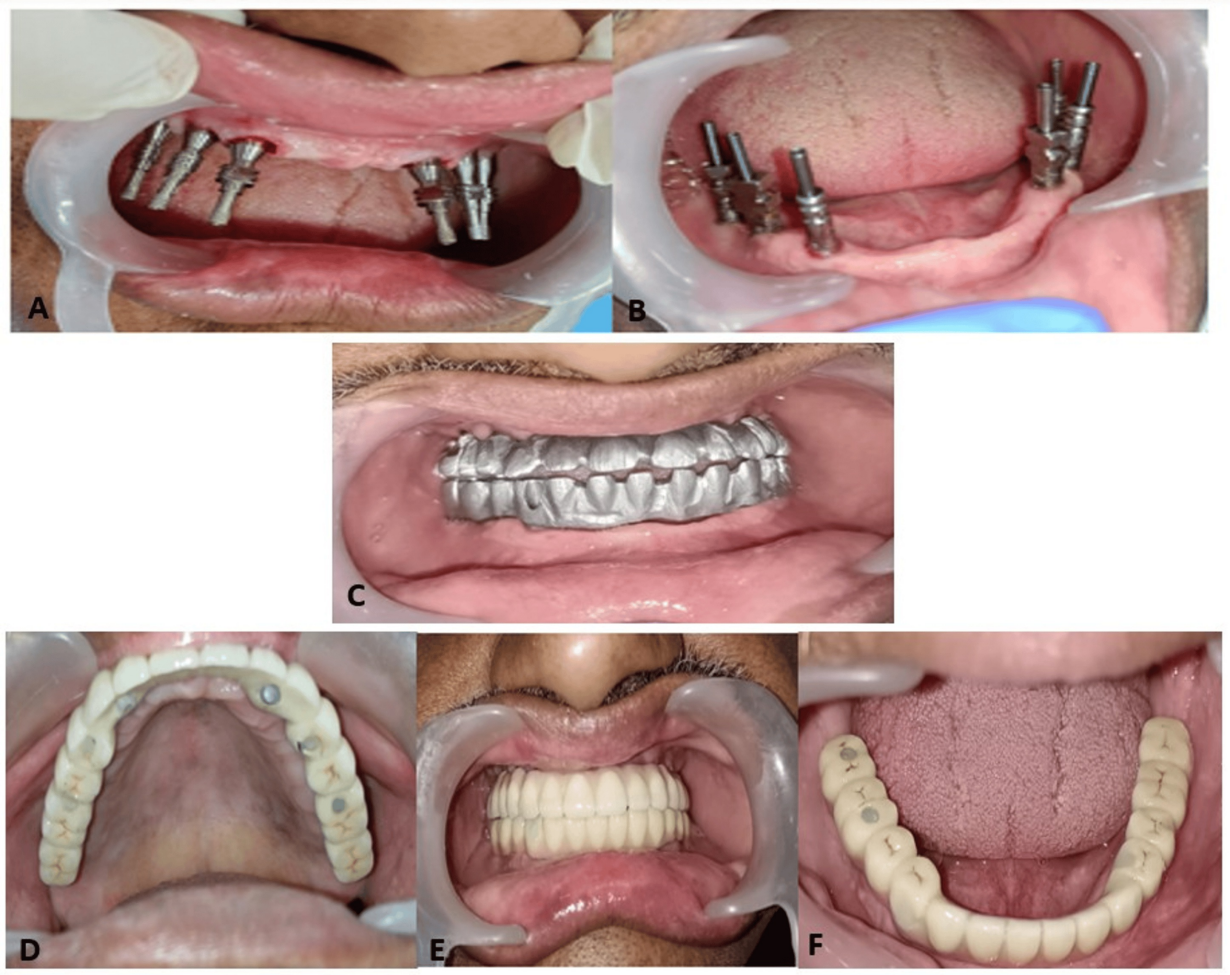
Sequential prosthetic workflow A-B: Open-tray impression copings connected to multi-unit abutments in the maxillary and mandibular arches, demonstrating accurate transfer of implant positions during impression making; C: Intraoral framework try-in to evaluate passive fit, marginal adaptation, and accuracy of the prosthetic framework; D-F: Definitive screw-retained full-arch implant-supported prosthesis in situ, illustrating occlusion, esthetics, and final prosthetic outcome

Following abutment placement, comfort caps were placed to protect the abutments and promote peri-implant soft tissue healing. After satisfactory soft tissue adaptation, open-tray impression copings were connected to the MUAs, and their positions were verified clinically (Figures 3A, 3B). A verification jig was fabricated using pattern resin to ensure accurate transfer of implant positions. The jig was sectioned and reluted intraorally where required to eliminate discrepancies and confirm passive fit. Passive fit was clinically verified using the Sheffield (one-screw) test and radiographic evaluation to ensure a strain-free and accurate prosthesis-implant interface. Subsequently, a definitive open-tray impression was made using a customized tray, ensuring precise reproduction of implant positions and surrounding soft tissues by using a polyvinyl siloxane elastomeric impression material.

The master cast was fabricated, and a metal framework was designed and cast. The definitive prosthetic framework was fabricated using a cobalt-chromium (Co-Cr) alloy to ensure adequate strength, rigidity, and long-term durability. A framework try-in was performed to evaluate passive fit, marginal adaptation, and accuracy of the prosthetic framework (Figure 3C). Adjustments were carried out as necessary to achieve a passive, strain-free fit. The definitive prosthesis was fabricated and delivered as a screw-retained full-arch implant-supported prosthesis. Final occlusal adjustments were performed to achieve harmonious occlusion with evenly distributed contacts. The prosthesis was secured by tightening prosthetic screws to the manufacturer-recommended torque, and screw access openings were sealed with composite resin (Figures 3D-3F).

A structured follow-up protocol was followed after prosthesis delivery, with recall visits scheduled at 24 hours, one week, one month, three months, and six months, and thereafter at six-month intervals. At each visit, peri-implant soft tissue health, prosthesis stability, occlusion, oral hygiene status, and patient comfort were evaluated. Minor occlusal adjustments were performed whenever necessary. At the six-month follow-up, radiographic assessment was carried out to evaluate peri-implant bone levels and implant stability. Marginal bone loss was minimal and within acceptable clinical limits (≤0.5 mm) during the six-month follow-up period.

Patient-reported outcomes were assessed using a visual analog scale, which demonstrated significant improvement in masticatory efficiency and overall satisfaction. Throughout the follow-up period, the patient exhibited satisfactory functional and esthetic outcomes, with healthy peri-implant tissues and no evidence of peri-implant radiolucency, implant mobility, or prosthetic complications.

## Discussion

The rehabilitation of completely edentulous patients using implant-supported full-arch prostheses has become a predictable and widely accepted treatment modality, providing superior functional efficiency, esthetics, and patient satisfaction compared to conventional removable dentures [11,12]. In the present case, a prosthetically driven approach was utilized to restore both arches with implant-supported fixed prostheses using MUAs, ensuring optimal biomechanics and long-term stability.

Achieving adequate primary stability and appropriate implant positioning is critical for successful osseointegration. In this case, osseodensification using Densah burs was employed in the maxillary arch to enhance bone density and improve implant stability in relatively low-density bone. This technique has been shown to preserve bone bulk and increase bone-to-implant contact, thereby improving primary stability and potentially enhancing clinical outcomes [13,14].

The placement of six implants per arch allowed for a favorable distribution of occlusal loads and a reduction of cantilever forces. This approach aligns with established full-arch rehabilitation concepts, such as the “All-on-4” and its modifications, which emphasize maximizing anterior-posterior spread and optimizing biomechanical performance [15,16]. Strategic implant positioning plays a crucial role in minimizing stress concentration and improving prosthesis longevity.

The use of MUAs was instrumental in correcting implant angulation and establishing a common path of insertion. Screw-retained prostheses supported by MUAs are preferred due to their retrievability and reduced risk of biological complications associated with residual cement [17]. Additionally, MUAs facilitate improved prosthetic accuracy and maintenance over time.

A delayed loading protocol with a healing period of five months was followed to ensure predictable osseointegration. Although immediate loading protocols have demonstrated promising outcomes, delayed loading remains a reliable and widely accepted approach, particularly in cases with variable bone quality or when optimal primary stability is desired [18]. This protocol minimizes micromovement at the bone-implant interface and enhances treatment predictability.

The open-tray impression technique combined with a verification jig ensured accurate transfer of implant positions and contributed to achieving a passive fit of the prosthetic framework. Passive fit is a critical factor in preventing mechanical complications such as screw loosening and framework fracture as well as biological complications, including peri-implant bone loss.

The structured follow-up protocol in this case demonstrated stable peri-implant tissues, the absence of inflammation, and no significant marginal bone loss. The patient reported marked improvement in masticatory efficiency and overall quality of life, which is consistent with previous studies highlighting the clinical success of implant-supported full-arch rehabilitations [12,19].

However, this report represents a single clinical case, and the findings cannot be generalized. Long-term follow-up and larger cohort studies are necessary to further validate the clinical outcomes and long-term success of such treatment approaches.

## Conclusions

Full-arch implant-supported rehabilitation using MUAs demonstrated a predictable and effective treatment outcome in the present single-case report of a completely edentulous patient. A prosthetically driven approach, strategic implant placement, and a delayed loading protocol were associated with favorable functional and esthetic outcomes in this case. The use of osseodensification in the maxillary arch appeared to enhance primary stability in areas of reduced bone density; however, this observation is based on a single case without a comparative control and should be interpreted cautiously. The treatment resulted in improved masticatory efficiency, patient comfort, and overall quality of life, with clinically stable peri-implant tissues observed during the follow-up period. However, although objective marginal bone-level measurements were included, the lack of long-term follow-up and comprehensive standardized outcome parameters remains a limitation of this report. Nevertheless, the absence of objective bone-level measurements and standardized outcome parameters represents a limitation. Therefore, while this approach showed promising results in the present case, the findings cannot be generalized, and further studies with larger sample sizes and standardized reporting are required to validate these outcomes.
